# Tales of me: storytelling identity work, authenticity, and impression management during new CEOs’ work role transitions

**DOI:** 10.3389/fpsyg.2023.1246887

**Published:** 2023-11-09

**Authors:** Bruno Felix, Renata dos Santos, Aridelmo Teixeira

**Affiliations:** Fucape Business School, Vitória, Brazil

**Keywords:** storytelling, identity work, new CEOs, macro work role transitions, grounded theory

## Abstract

**Introduction:**

This study aims to understand how new CEOs construct, revise, and maintain in their narrative, repertoire stories that represent their identity as it is associated with their new occupational role.

**Methods:**

For this purpose, we conducted a qualitative study using the Grounded Theory method, involving 47 CEOs from different sectors residing in Brazil.

**Results:**

Our results allowed for the construction of a central category called Storytelling Identity Work, which refers to stories that individuals tell about themselves, and enable them to propose and redefine who they are during major life transitions. This paper seeks to explain: (a) under what conditions this phenomenon tends to occur; (b) what characterizes the success of Storytelling Identity Work and; (c) what leads to the revision or retention of stories in the self. Our results show that storytelling identity work tends to be used by new CEOs during their transition period into the role, and when their new position involves higher levels of visibility and alteration of prestige levels in comparison to their previous position. They also suggest that storytelling identity work tends to be more successful when the stories are co-constructed and validated with other significant individuals and when they enable new CEOs to feel “sufficiently authentic” and “sufficiently impressive.” Finally, we theorize that such feelings, along with a sense of coherence between the story being told and other narratives consciously or unconsciously being narrated by the interviewees throughout their lives, lead to the retention of the story within the individual’s self.

**Discussion:**

This article innovates by connecting the literature on personal storytelling with identity work and exploring processes that are not only useful during the process of transitioning into the role of new CEOs but also influence the constitution of the narrative repertoire and, consequently, the identity of these individuals.

## 1. Introduction

The phenomenon of CEO succession has been extensively explored in the finance and strategy literature due to its critical role in organizational success (Krishnan Kozhikode and Krishnan, 2022; Nguyen and Lee, 2023). However, little attention has been given to how new CEOs navigate the identity transition processes involved in their succession (Yi et al., 2020). The arrival of a new CEO in this hierarchical position can be considered a macro work role transition (Sergeeva and Green, 2019), in which they will need to negotiate and manage a series of “who am I” aspects, such as clothing, workplace decoration, and even the language they use (Felix and Cavazotte, 2019; Kulkarni, 2020). One way in which individuals manage their identities in macro work role transitions involves creating narratives, stories, and other rhetorical resources through which they seek to construct new meanings for themselves and others about who they are (Sergeeva and Green, 2019). These elements need to align with the expectations of the new roles they assume in life, and in the case of new CEOs, these expectations tend to be extremely challenging (Quigley et al., 2019). It is not uncommon for new CEOs to struggle to feel or be socially validated as possessing the characteristics expected of someone in this position, which can be detrimental to their success in the role and, consequently, to the company that hired them (Cowen and Montgomery, 2020). However, despite the importance of identity work for new CEOs, further research is still needed on the rhetorical self-constructions they develop when assuming this position (Maclean et al., 2021; Srivastava et al., 2023).

Rhetorical self-constructions are self-narratives that seek to propose, express, and constitute an identity (Ibarra and Barbulescu, 2010; Wood et al., 2021). In this work, identity is understood as “the internalized and evolving story that results from a person’s selective appropriation of past, present and future” (McAdams, 1999, p. 486). Studies have indicated that rhetorical self-constructions are used by individuals to cope with identity threats, and to propose desired changes in their self-concept, especially when they assume new roles in life (Ezzy, 1998; Ibarra and Barbulescu, 2010; Rostron, 2022). However, it is still necessary to better understand how specific stories are discarded for being deemed implausible or retained to be told more frequently in the future, thus forming a plot, or repertoire, of stories that enable the construction of a desired new identity (Cinque et al., 2021).

This view of identity as rhetoric is particularly relevant in macro role transitions, which are moments of passage that individuals experience between organizational, professional, occupational, or personal roles (George et al., 2022). Examples include becoming a CEO, becoming a parent, or undergoing a drastic career change (Göktürk et al., 2020; Felix et al., 2023b). This concept differs from that of micro role transitions, which involve psychological and physical movements within the same role (Ashforth et al., 2000) such as transitioning between working from home and working in an in the same day. CEO macro role transitions are challenging because they often require leaders to construct a new “self” for their new role that meets expectations often close to those of a celebrity (Lee et al., 2020), but one that also allows them to feel authentic (Ladge et al., 2012; Hennekam and Ladge, 2022), and is considered plausible and validated by other significant individuals (Cragun et al., 2020). Thus, the stories that new CEOs tell about themselves in this transition process are simultaneously relevant and risky (Kibler et al., 2021). This is because while some may engage in self-promotion in their narratives, others may aim to construct a more authentic self that does not live up to the expectations associated with their new role (Byrnes and Taylor, 2015). Additionally, it is necessary to better understand how these stories evolve over time, how they are validated by the individual and others, and how they are retained in their self-narrative repertoire or discarded (Castelló et al., 2023).

In the face of these knowledge advancement opportunities, the objective of this study is to understand how new CEOs construct, revise, and maintain stories in their self-narrative repertoire that represent their identity as associated with their new occupational role. For this purpose, inspired by Social Identity Theory (Tajfel and Turner, 2004), we conducted a qualitative study using the Grounded Theory method, involving 47 CEOs from different sectors, all of whom were residing in Brazil. The results led to the construction of a central category called storytelling identity work, which refers to stories that individuals tell about themselves and enable them to propose and redefine who they are during major life transitions. This work sought to explain: (a) under what conditions this phenomenon tends to occur, (b) what characterizes the success of storytelling identity work, (c) what leads to the revision or retention of stories in the self. This article has theoretical implications for literature exploring the relationship between role transitions and identities (Currie et al., 2010; Hennekam, 2016; Bordia et al., 2020). The concept of identity work has already been well-established in the literature, and numerous studies have investigated how individuals engage in processes of constructing and revising their selves in the workplace (Li et al., 2023; Sentuti et al., 2023; van Merriënboer et al., 2023). It is also known that this trial-and-error process in the pursuit of constructing provisional selves through storytelling entails risks, while being fundamental for individuals’ career advancement (Byrne et al., 2021; Corlett et al., 2021). However, this article innovates by connecting the literature on personal storytelling with identity work and exploring processes that are not only useful during the process of role transition as new CEOs but also influence the constitution of the narrative repertoire and, consequently, the personal identity of these individuals (Ibarra and Barbulescu, 2010; Bloom et al., 2021). Thus, this study responds to the call for articles that adopt a processual view of identity phenomena in the workplace (Haynie and Shepherd, 2011), as identities have become increasingly less predictable and labor markets have been requiring greater adaptability from individuals (Mansur and Felix, 2021).

## 2. Literature review

### 2.1. Identities and identity work: a narrative perspective

According to Social Identity Theory (Tajfel and Turner, 2004), identities refer to distinct meanings ascribed to an individual by both themselves and others (Ashforth and Schinoff, 2016). These constructions about the self can refer to social roles that individuals perform (social identity) or to personal characteristics and behaviors that confer personal distinctiveness to certain individuals (personal identity) (Ashforth and Humphrey, 1995; Ozyilmaz and Koc, 2022). Identities do not only reflect the lived self but also the desired self, in such a way that it can be understood as multiple, transient, and (re)constructed through social interactions (Ashforth and Mael, 1989). At times, these self-definitions may conflict with each other, but they tend to coexist and integrate into units that provide individuals with a certain emotional and cognitive stability (Petriglieri, 2011).

During the process of socialization and individuals assuming new roles, such as CEOs, individuals tend to negotiate “who I am” in the face of “who I am expected to be” (Felix and Cavazotte, 2019; Felix et al., 2023a). Both individuals and the social expectations associated with certain work roles are dynamic, which means that the relationship between them also evolves over time (Kreiner et al., 2006). The agentic responses that individuals offer to these dynamic interactions characterize “identity work,” which encompasses “a range of activities in which individuals engage to create, present, and sustain personal identities that are congruent with and supportive of the self-concept” (Snow and Anderson, 1987, p. 1348). This process involves the formation, alteration, maintenance, revision, or reinforcement of constructions about the self that generate a sense of uniqueness and coherence (Sveningsson and Alvesson, 2003). It can occur through mechanisms such as rhetorical self-constructions for the experimentation of possible identities, including self-presentation on online social networks (Rothbard et al., 2022), self-narratives (Ibarra and Barbulescu, 2010), and corporate discourses (Heinrichsmeier, 2021), for example. This study focuses on a specific form of identity work: the stories that individuals tell about themselves.

Rhetorical self-constructions are self-narratives that aim to propose, express, and constitute an identity (Ibarra and Barbulescu, 2010; Wood et al., 2021). In this study, we rely on the understanding that an identity is formed by “an internalized story that changes over time, resulting from a selective elaboration individuals make about the events they have experienced, are experiencing, and will experience” (McAdams and McLean, 2013). Although there are different definitions of storytelling, we define it here as a meaning-making process about oneself through the narration of lived events (Sims, 2003). By telling stories about themselves, individuals express, construct, and transform their identities, and, for this reason, storytelling can be considered a form of identity work (Alvesson, 2010). However, since a better understanding of the conditions under which this form of identity work is adopted by new CEOs is still needed, we present our first research question (RQ):

RQ1: When do new CEOs tend to use storytelling to perform identity work?

### 2.2. New CEOs’ role transitions and identity work

The literature on identity transitions has focused on both macro and micro role transitions. While macro transitions refer to significant shifts that individuals experience between organizational, professional, or occupational roles (George et al., 2022), micro transitions involve psychological and physical movements within the same role (Ashforth et al., 2000). Assuming the position of a CEO can be considered a macro role transition as it often involves a reconstruction of one’s image in order to be seen as capable of fulfilling this new position (Barnes, 1988; Bach and Serrano-Velarde, 2015). Whether it’s an executive promoted for the first time to the CEO position, or someone who has previously held the role in another organization, new CEOs inevitably engage in self-reflection processes that require identity work (Byrnes and Taylor, 2015; Bojovic et al., 2020).

The macro identity transitions experienced by new CEOs present significant challenges for these individuals in terms of the need to simultaneously feel authentic in their new role and impress their audience (Guthey and Jackson, 2005). Although there are various approaches to authenticity in the workplace, the dominant view is that it involves a congruence between individuals’ external and internal identities (Caza et al., 2018; Hewlin et al., 2020; Bailey and Iyengar, 2022; Boytos et al., 2022). When expressed, authenticity is often reported as “authentic self-expression,” “feeling authentic,” “authentic behavior,” and “being authentic” (Cha et al., 2019, p. 634). These views implicitly suggest that individuals are aware of how to express their self, while recent evidence challenges this notion (Wilt et al., 2019; Hennekam and Ladge, 2022; Conde et al., 2023). According to this emerging perspective, individuals’ sense of self evolves over time, and therefore, the expression of a “true self” would be a utopia (Peterson, 2005). This perspective, which seeks to understand “becoming authentic” rather than “being authentic” (O’Neil et al., 2020), needs to be explored in more detail. It is necessary to take into account the specificities of identity transitions that have a greater impact on individuals’ lives (Hennekam and Ladge, 2022).

Parallel to its implications in terms of authenticity, the macro role transition experienced by new CEOs also creates a need to manage the impressions others have of them (Graffin et al., 2011). For instance, prior evidence suggests that these professionals tend to dedicate themselves to expressing themselves in socially desirable ways in strategic presentations (Whittington et al., 2016; Collewaert et al., 2021), letters (Boudt and Thewissen, 2019), financial reports (Bujaki and McConomy, 2015), and online social networks (Zhang and Mooney Murphy, 2021) as means to construct a positive image among relevant stakeholders. However, this process is not without risk, as excessive efforts can lead to irreversible damage to their reputation (Usmani et al., 2020). Therefore, it is necessary to gain a better understanding of the limits of new CEOs’ efforts to build a social image commensurate with their new roles (Verhaal and Dobrev, 2022) and comprehend what characterizes an identity work as successful. It is essential to explore a perspective that goes beyond binary thinking and theorizes about the paradoxes experienced by new CEOs as they seek to express themselves and influence others (Ibarra, 2015; Verhaal and Dobrev, 2022). Thus, we present our second research question:

RQ2: What characterizes the success of the identity work accomplished through storytelling?

In general, individuals tell different stories about themselves as they present their self to others (Bateson, 2004). They create and reinterpret the stories they choose to tell regarding the events they experience (Boje, 1991). Some of these stories are frequently repeated and shared with different audiences, or even with the same audience at different times, depending on individuals’ objectives with these processes (Ryan, 2008). Thus, individuals construct not only isolated stories but also a repertoire of stories that are more or less coherent with each other and tend to be told as a means of managing their identities (Ibarra and Barbulescu, 2010). When telling their stories, individuals test different strategies in terms of style, attitudes, language, humor, and intonation, for example, which lead to different dramatic effects (Sharma and Grant, 2011). Some of these strategies are seen as more successful for identity work goals and become incorporated into the personal narrative repertoire and, consequently, into personal identity (Zilberstein et al., 2023). However, others tend not to be retold because they did not achieve their initial objective and are omitted and discarded from the self-construction process (Ibarra and Barbulescu, 2010). This process is still not well understood concerning individuals in general and even less so in the specific case of CEOs, despite their identity transitions being relevant to the future of many organizations (Bloom et al., 2021). Thus, we arrive at the third research question for the grounded theory developed in this study:

RQ3: What leads stories to be revised or retained in the self?

## 3. Methods

The present qualitative study was conducted using the Grounded Theory method (Gioia et al., 2013). The choice of this method was due to the lack of previous studies exploring the phenomenon of our interest from a storytelling perspective. Aligned with the principles of Grounded Theory, the decision to present theories in the previous section is because, in this method, it is important to present concepts and theories that, although not serving as foundational theories, act as sensitizers for immersion in the field (Bowen, 2006; Felix and Cavazotte, 2019; Felix et al., 2023c). The Grounded Theory method allows for the proposition of a theory through an iterative process of data collection and analysis (Charmaz, 2014). This process aims to construct a model that allows for parsimonious theorizing about the concepts that explain the phenomenon in question, their properties, and the relationships between the categories that occupy a central position in its explanation.

In line with the principles of Grounded Theory (Glaser and Strauss, 2017), the selection of research participants was based on theoretical sampling criteria. Initially, we sent an invitation to a list of 545 CEOs residing in Brazil who were clients of a leadership training company. Of these, 24 responded positively and made themselves available for interviews. From this initial list, 8 individuals were interviewed, constituting the first wave of data collection. In the second round, we interviewed another 8 CEOs, and the same happened for the third wave of data collection. As we observed that new categories were still emerging after these three rounds, we chose to employ the snowball sampling technique (Naderifar et al., 2017; Felix et al., 2018) to access new CEOs. To achieve this, it was necessary to employ relational skills so that the interviewed executives would trust us enough to recommend new CEOs who could participate in the research. This process led to the identification of an additional 31 potential interviewees. Thus, in the fourth wave of data collection, 12 of these individuals were interviewed, and in the fifth and final wave, where data saturation was reached, we collected data from another 11 CEOs.

Thus, the final sample consisted of 47 CEOs. Table 1 presents information on the demographic profile of the research participants and the companies they work for. The participants had an average age of 54.1 years and are predominantly male (85.1%), White (91.5%), Brazilian (89.4%), and work in the following sectors: industrial (7), financial (6), cyclical consumer goods (5), public utilities (5), healthcare (5), energy (4), materials (4), non-cyclical consumer goods (4), information technology (3), real estate (3), and communication services (1). Most interviews were conducted in the Portuguese language (89.4%), with 53.2% conducted in person, while the remaining 46.8% were conducted remotely at the request of the interviewees. The average duration of the interviews was 49.2 min, and their contents were recorded and transcribed.

**Table 1:** Demographic profile of participants.

**Code**	**Gender**	**Age**	**Race**	**Nationality**	**Interview language**	**Previous CEO experience?**	**Interview duration (in minutes)**	**Interview type**
E1	Male	68	White	Brazilian	Portuguese	No	38	In person
E2	Male	53	White	Brazilian	Portuguese	No	39	In person
E3	Male	39	White	Brazilian	Portuguese	No	72	Remote
E4	Male	50	White	Brazilian	Portuguese	Yes	79	Remote
E5	Female	58	White	Brazilian	Portuguese	Yes	60	In person
E6	Male	65	White	Brazilian	Portuguese	Yes	45	Remote
E7	Male	51	Brown	Brazilian	Portuguese	Yes	38	In person
E8	Male	54	White	Brazilian	Portuguese	No	49	Remote
E9	Male	46	White	Brazilian	Portuguese	No	53	In person
E10	Male	55	White	Brazilian	Portuguese	No	58	In person
E11	Male	55	White	american	English	Yes	50	Remote
E12	Male	61	White	Brazilian	Portuguese	No	46	Remote
E13	Male	40	White	Brazilian	Portuguese	No	45	In person
E14	Female	57	White	Brazilian	Portuguese	Yes	71	In person
E15	Male	42	White	Brazilian	Portuguese	No	38	In person
E16	Male	41	White	Brazilian	Portuguese	No	62	Remote
E17	Male	47	Brown	Brazilian	Portuguese	Yes	61	Remote
E18	Male	54	White	Brazilian	Portuguese	No	52	Remote
E19	Male	56	White	Brazilian	Portuguese	No	35	In person
E20	Male	52	White	Brazilian	Portuguese	No	37	Remote
E21	Male	55	White	Brazilian	Portuguese	Yes	67	In person
E22	Female	62	White	Brazilian	Portuguese	No	53	In person
E23	Male	59	White	American	English	Yes	38	In person
E24	Male	65	White	Brazilian	Portuguese	No	51	Remota
E25	Male	63	White	Brazilian	Portuguese	No	58	Remote
E26	Male	56	White	Brazilian	Portuguese	No	64	In person
E27	Male	63	White	Brazilian	Portuguese	No	56	In person
E28	Male	52	White	Brazilian	Portuguese	No	39	In person
E29	Female	55	White	Brazilian	Portuguese	Yes	55	Remote
E30	Male	61	White	Brazilian	Portuguese	No	40	In person
E31	Male	48	White	Brazilian	Portuguese	No	39	Remote
E32	Male	53	White	Brazilian	Portuguese	No	41	Remote
E33	Female	47	White	Brazilian	Portuguese	Yes	51	Remote
E34	Male	49	White	Brazilian	Portuguese	No	39	In person
E35	Male	45	White	British	English	Yes	57	In person
E36	Male	57	Black	Brazilian	Portuguese	No	43	Remote
E37	Male	62	White	French	English	Yes	38	In person
E38	Female	56	White	Brazilian	Portuguese	No	45	Remote
E39	Male	62	White	Brazilian	Portuguese	No	49	In person
E40	Male	54	White	Brazilian	Portuguese	No	43	In person
E41	Male	49	White	Brazilian	Portuguese	Yes	46	Remote
E42	Male	39	White	Chinese	English	Yes	33	Remote
E43	Female	56	White	Brazilian	Portuguese	Yes	65	In person
E44	Male	45	White	Brazilian	Portuguese	No	38	In person
E45	Male	71	Brown	Brazilian	Portuguese	Yes	51	Remote
E46	Male	61	White	Brazilian	Portuguese	No	39	Remote
E47	Male	55	White	Brazilian	Portuguese	No	45	In person

The interviews were conducted using a semi-structured protocol that, in line with the recommendations of the Grounded Theory method, was continually refined throughout the five rounds of data collection. The main challenge in conducting the interviews was the scarcity of interviewees, which we sought to address through an objective approach to data collection. The final version, which guided the fifth round of interviews, is presented in Table 2. The interviews focused on the first year in which the interviewed executives served as CEOs of their current company.

**Table 2:** Final interview protocol.

1. Tell us about yourself and your professional journey so far.
2. Please share the process that led you to achieve the position of CEO. Have you held this position before? How was the transition process to this new role in your life, considering your experience in this company?
3. How was the specific period between receiving the invitation for this position in this company and the subsequent 12 months after starting your tenure as CEO?
4. These questions and the subsequent ones will refer to the period between receiving the invitation for this position in this company and the subsequent 12 months after starting your tenure as CEO. What stories do you often tell about yourself when introducing yourself in personal and professional life? In which occasions do you find these stories being told more frequently or inhibited from being shared?
5. Why do you believe you tell such stories? In what way do they assist you? Have they ever hindered you? If so, how and why did that happen?
6. JHave there been stories about yourself that you have chosen not to tell for some reason? If so, what was the reason for that?
7. Why, in contrast, do you think other stories ended up being told more frequently?

To analyze our data, we utilized a two-step coding approach reminiscent of the one employed by Kreiner et al. (2009). Initially, we created inductive codes based on the data, treating every phrase and word as potential data units eligible for coding (Heath and Cowley, 2004; Glaser and Strauss, 2017). During this initial phase, we documented all first-order codes within an emerging codes lexicon (Charmaz, 2014). Subsequently, two of the three authors independently reviewed the interview excerpts supporting each code and aimed to cluster them under the initially established first-order codes, akin to prior research (Mace and Ward, 2002; Stough and Lee, 2021). The coders had the freedom to assign data segments to pre-existing codes or exclude them. In cases of disagreement, an impartial adjudicator assessed the interview excerpt, fostering opportunities for theory development through reflective discussions about the data. This iterative process transpired after each data collection wave, allowing us to enhance the code lexicon. Thus, our coding process was characterized by multiple perspectives, mitigating potential interpretational biases (Kolb, 2012).

**Figure 1:**
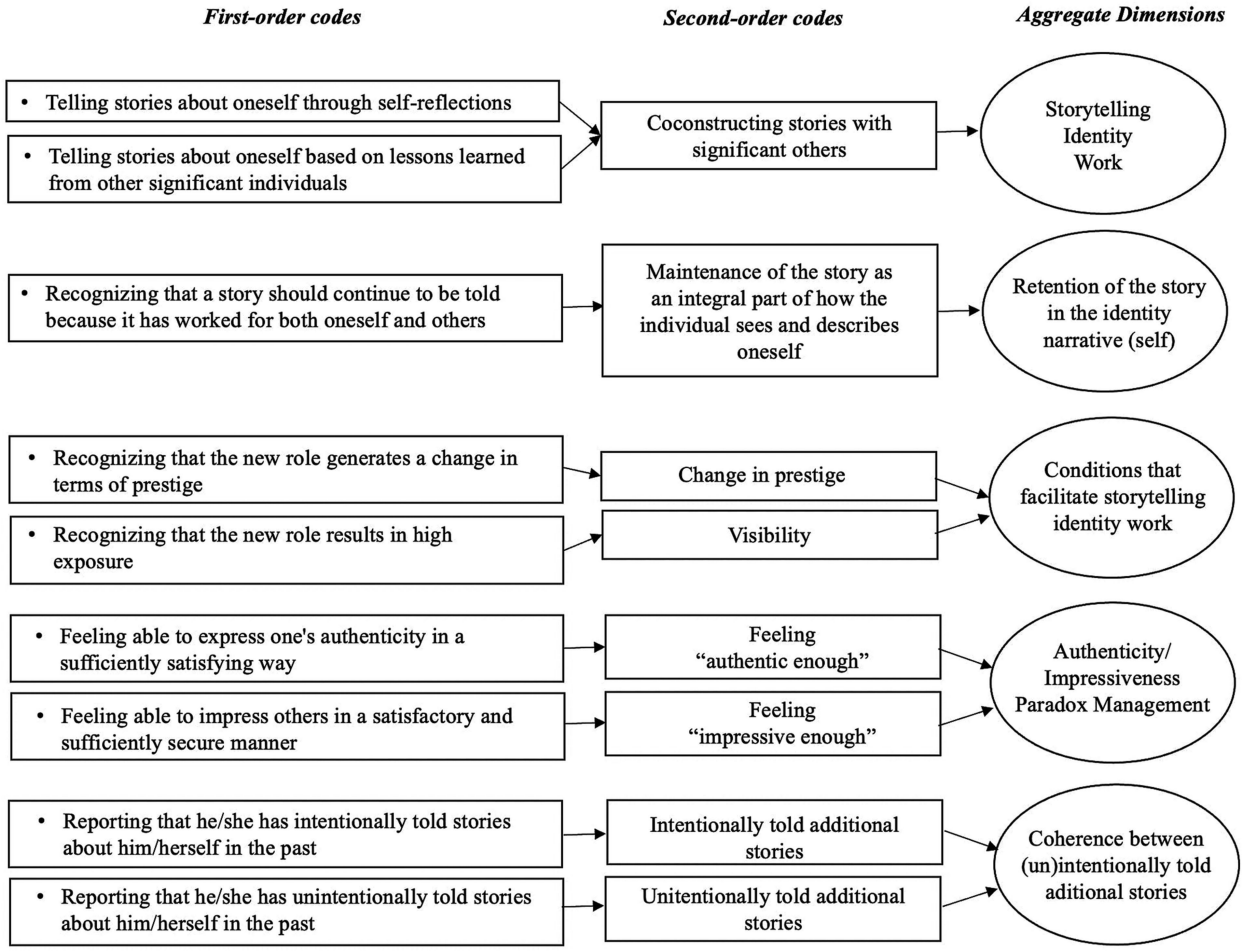
Data structure.

Once the first-order codes achieved greater stability, we proceeded to the analysis with the objective of categorizing them into more abstract codes referred to as second-order codes. The same coding verification process, involving two authors and subsequent evaluation by an impartial adjudicator, was employed to derive second-order codes from our inventory of first-order codes (Nag and Gioia, 2012). After the second-order codes also exhibited increased stability, we replicated the same procedure to consolidate them into aggregated dimensions (Gioia et al., 2013), representing even more abstract codes with broader theoretical significance. This approach aligns with other grounded theories (Byron and Laurence, 2015; Felix and Cavazotte, 2019). The overall agreement percentage between the two coders stood at 0.92, surpassing the recommended minimal threshold of 0.70 proposed by Cohen (1960) and Kreiner et al. (2009). In Figure 1, we present the data structure resulting from the data analysis process, elucidating the progression from first-order codes to aggregated dimensions.

Ultimately, the categories with greater theoretical scope (second-order codes and aggregated dimensions) were gathered in a model (Chandra and Shang, 2019) that sought to answer the three central research questions outlined in the literature review. It is important to emphasize that these three research questions were not developed *a priori*: they emerged during the process of data collection and analysis. It means that the literature review was conducted after the creation of the grounded theory presented here.

## 4. Research context

Brazilians are among the most active users of social media worldwide (Silva et al., 2023). According to research, Brazil has one of the highest rates of social media penetration in its population, with over 140 million active users (Statista, 2021). This strong presence on social media has a significant impact on how people relate and construct their online identities, including executives and CEOs. In this scenario, it is common to observe the valorization of the celebrity status of Brazilian executives, especially on social media. This tendency is further exacerbated by being a country with high power distance levels (Hofstede, 2011; Felix et al., 2019), which encourages individuals to display power and authority to meet the social positions they hold (Hofstede et al., 2010). An emblematic example is the case of Bel Pesce, an entrepreneur and former executive in prominent companies. Her stories about her career and achievements were widely disseminated on social media, and she became an influential figure in Brazil’s entrepreneurial scene. However, her narratives were subsequently contested and publicly questioned, raising debates about her credibility and authenticity (Carvalho, 2019). This case illustrates how the stories shared by CEOs can be subject to scrutiny, especially in a context where exposure on social media is high and in cultures where there is a high power distance, as is the case in Brazil (Hofstede et al., 2010; von Borell de Araujo et al., 2014).

## 5. Results

**Figure 2:**
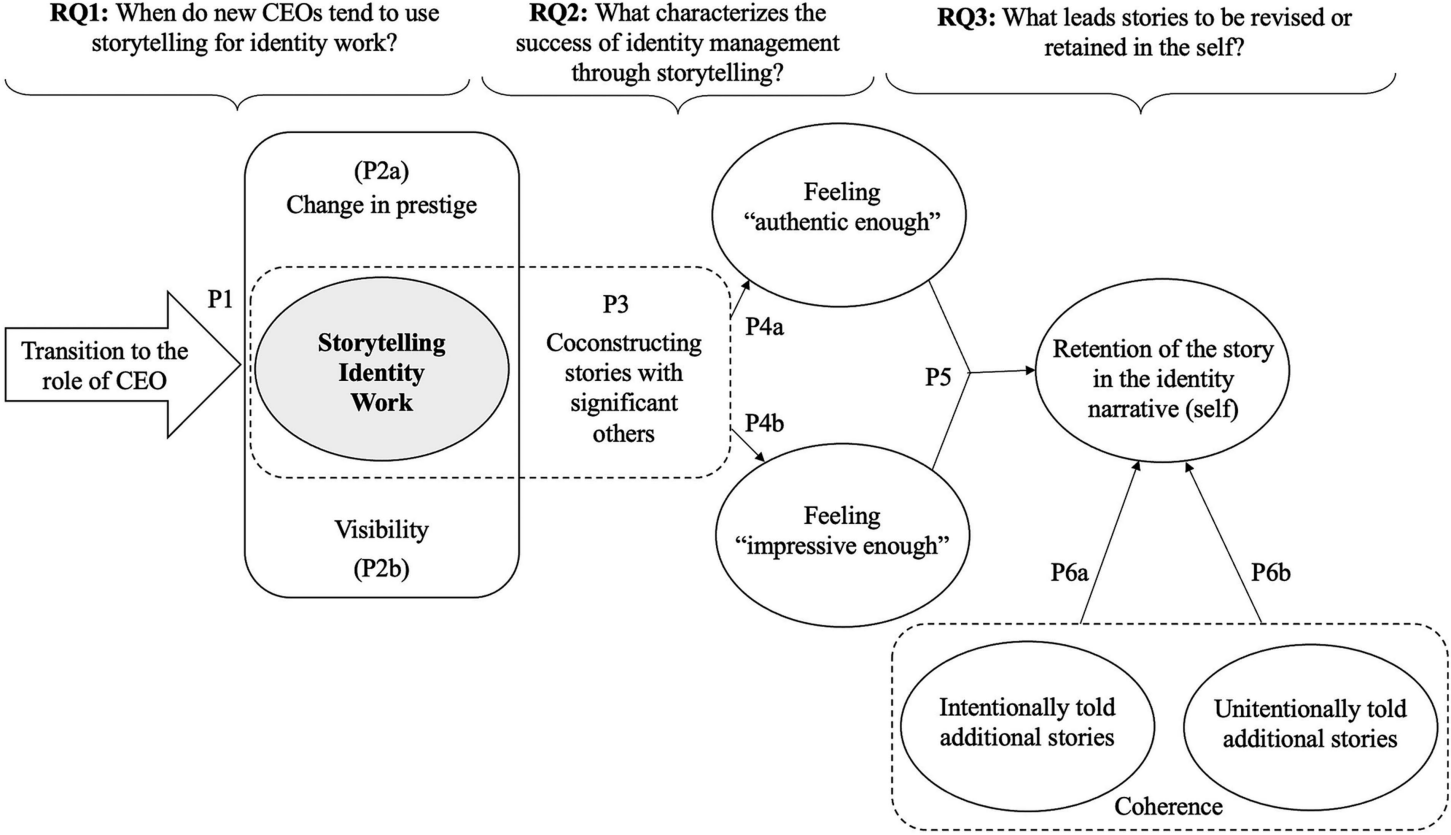
A model on storytelling identity work by new CEOs.

In this section, we present the results of this study in the form of a model, which was built based on a set of theoretical propositions. The model addresses three central research questions (RQs): (a) the circumstances that facilitate the use of storytelling for conducting identity work (RQ1, Propositions 1 and 2); (b) what characterizes the success of storytelling identity work (RQ2, Propositions 3 and 4); and (c) what leads to the revision or retention of stories in the self (RQ3, Propositions 5 and 6). To make the writing more parsimonious, hereafter the propositions will be represented by the letter “P” (P1 to P6a and P6b). Figure 2 presents a visual representation of the model and the propositions that compose it. We detail the presentation and explanation of the model, as well as the empirical evidence supporting it, in the following three topics, structured based on the three research questions from which we constructed our model.

### 5.1. When do new CEOs tend to engage in storytelling identity work?

To address our first research question, we sought to understand how the CEOs interviewed dealt with the months following the macro transition to the role associated with this new professional challenge. Several CEOs argued that upon becoming CEOs, they had to redefine their conceptions of who they are through actions such as decorating their workspace with “objects that symbolize greater power, like more expensive pens” (I5), “personal photographs showcasing past professional achievements” (I32), and dressing in “more expensive clothing that are typically worn by other CEOs” (I15). Others, on the other hand, reported adopting daily rituals such as “posting pictures of morning coffees with directors” (I9), “reading newspapers in the morning accompanied by technical analysts” (I23), and “posting pictures on social media at events with other CEOs.” For many interviewees, these actions “help rebuild the notion of who we are because we need to believe in ourselves and convince others that we are worthy of being a CEO” (I10). However, some interviewees also mentioned that, in addition to these forms of identity work, they began to share specific stories about their personal and professional journeys that positioned them as individuals deserving of the CEO role. This phenomenon, referred to here as “storytelling identity work,” is evidenced in the following statements from the interviewees:

"I noticed that, during this period after assuming the CEO position, I kind of revisited my past and redesigned certain stories that I had lived. I learned to tell stories in a more engaging way because I know it inspires and helps to gain people's trust. And certainly, I started doing it much more during this period than before and after I got accustomed to the new position" (I12).

"Well, when I took on the role, I thought to myself: man, I'm just an ordinary person. All the CEOs I've had throughout my career were super full of stories, always telling stories that made people laugh, that impressed people, that projected that image of 'I'm awesome.' So yes, I admit that I found myself thinking, reflecting on how I would tell certain stories in everyday situations, while others emerged spontaneously. And after that first year period passed, I feel like I kind of stabilized that, just maintaining the stories I had built during that initial period" (I29).

It cannot be denied that the evidence shows that the interviewees adopted the practice of storytelling with the aim of managing their identities at work both before and after transitioning to the CEO role. However, our data indicate that the phenomenon of storytelling identity work was more intense during the months of transition. According to some interviewees, assuming the CEO position even involved hiring a “speech and personal storytelling specialist for executives” (I32) and a “coach to help build more engaging stories” (I46) about themselves. Outside of the transition period, there were more frequent revisions to existing stories and a “lower effort in terms of considering how to present oneself” (I1). In light of these findings, we propose that:

Proposition 1: Storytelling identity work is more frequently adopted by new CEOs during the transition process to this identity than outside of this period.

The individual experiences during the transitions to the CEO position differed among the interviewees. Two factors related to the nature of the transition stood out during the data analysis: the presence of higher or lower levels of prestige and visibility involved in the transition process. The levels of prestige varied among the interviewees primarily due to the fact that, while for some individuals who had previously held CEO positions in organizations with greater reputation, the appointment to the position represented a career downgrade, while, for others, it signified reaching a new career level. In both cases, whether to demonstrate purpose in accepting the new role or to prove oneself worthy of the CEO position, the interviewed individuals showed greater engagement in the use of storytelling identity work compared to those for whom the new appointment did not entail a significant change in their previous level of prestige. The following evidence supports this understanding.

"I had previously been the CEO of [leading company in the sector, name withheld]. I took a sabbatical year and now I reappear here, at the fourth-ranked company in the industry. Everyone knows it was a downgrade in my career because I was fired from there. But it was obvious that it bothered me, people could look at me and think that I was entering here because the value of my worth had decreased, that I wasn't as good. So, I ended up telling a whole story of career realignment, recounting episodes of involvement of that company in corruption that were publicly known to justify the change. It did influence, but the way I tell it, I make it seem like I chose to leave and not that I was forced out" (I45, example of “high prestige reduction”).

"I went from being the Marketing Director to CEO in a very short period of time and at a young age. This would cause a great deal of strangeness among people, especially because I am a woman. So, I stopped and had to think about a whole set of stories, about how I would present myself, especially in the TED Talk I gave. I needed to appear up to the task, like a prodigy woman. So, I not only hired a personal stylist, but also a consultant to help me present myself, to retell how I talk about myself, my background, my accomplishments" (I38, example of “high prestige increase”).

"I followed the natural succession line within the company, everyone saw the signs in the past two years that I would take on [the CEO position]. So, I don't know, it was a more natural process. I did find myself reconsidering my story, retelling some things, choosing my words, concealing certain parts, highlighting others. When it happens, it's always a shock. But I don't know, talking to other CEO friends, I noticed that I didn't use this resource as much as they did" (I39, “irrelevant prestige change”).

The evidence presented demonstrates that, in the first two cases, where there was, respectively, a loss and a gain of prestige upon assuming the new CEO position, the interviewees relied more on the use of storytelling identity work compared to the third case, where the new position did not alter the individual’s reputation as much. Thus, we observed that they perceive a greater need to revisit the way they present themselves through self-narratives when they perceive a gap between how they are interpreted (perceived identity) and how they would like to be perceived (desired identity). Therefore, we theorize that:

Proposition 2a: The greater the perceived variation in prestige level after transitioning into the identity of new CEOs, the more frequently these individuals adopt storytelling identity work.

Another factor that influenced the use of storytelling identity work was the visibility of the new CEO position. Some executives assumed roles in companies that “do not rely heavily on marketing to build relationships with stakeholders” (I40), while others stated that they “need to be visible all the time” (I39). For instance, a CEO of a company providing electricity supply services mentioned, “My position is not sexy, nobody cares about who the CEO of [company name] is. But when I was the CEO of [name of a retail company], I was a pop star; the company had a progressive positioning, and I had to have stories aligned with that” (I41). Another interviewee reported:

"My company is in the fashion industry, and we take a stance on various controversial topics. I am often invited to startup events as an experienced executive, so becoming a CEO required a level of visibility that demanded me to revisit the way I talk about my story, about myself" (I14).

Thus, we observed that, according to the interviewees’ accounts, although the CEO position generally leads to high personal exposure, the degree of visibility, which refers to the extent to which individuals are observed by stakeholders, varied in the experiences of the interviewees. The data showed that when visibility was higher, individuals felt more pressured to meet their audience’s expectations with stories that positioned them in line with the perceived expectations placed upon them. On the other hand, those who reported being less observed expressed a lower need to tell stories to manage their identities. Based on these findings, we suggest that:

Proposition 2b: The higher the level of visibility involved after transitioning into the identity of new CEOs, the more frequently these individuals engage in storytelling identity work.

### 5.2. What characterizes the success of storytelling identity work?

To address the second research question that guided the development of this grounded theory, we sought to inquire with the interviewees about what they consider to be a successful storytelling identity work. Consistent with the intra- and intersubjective nature of identities, several interviewees reported that a successful story is one that “you feel that you and others believe in” (I47). In some cases, interviewed CEOs mentioned that “some stories did not resonate well, they seemed exaggerated, neither I nor anyone else bought into them, and I see that as a story that was not successful” (I38). Thus, intra- and intersubjective validation was considered the primary criterion for defining, based on the participants’ accounts, what constitutes a successful story.

The first factor identified as leading to this perception of intra and intersubjective validation was the interaction with other CEOs as well as additional significant members of the audience in the process of constructing stories. According to the statements from several interviewees, storytelling identity work is not only a process of presenting content to an audience but also a process of co-construction. This occurs especially in the interaction with individuals of high prestige or who currently or previously held the same role, even in other companies. The following accounts provide evidence for this finding:

"I noticed that when I shared my story with another CEO and they shared theirs, whether in conversations or at events, I could see that some things were similar, while others stood out, eliciting grimaces or being ignored. I intuited this and naturally began filtering and adjusting my story to make it more aligned with theirs. Their reactions and borrowing the moral of the stories they told became key for me to understand what needed to be adjusted. As I did this, I realized that the stories I tell about myself improved, I became more persuasive, and I also convinced myself in the process" (I19).

"I didn't have the right approach when it came to crafting my own stories. Within a week of assuming the position, I realized that my way of telling my story wasn't effective; it was too low profile. My story seemed too trivial. I began to notice that everyone embraced the Hero's Journey, that nonsense that everyone enjoys and that I saw commanded respect. So, I started testing an exaggerated way of narrating certain events—a crisis, a relationship with a mentor, a great battle I won, and so on—and I observed that the story became more intriguing and successful for what I needed. I observed the reactions of my peers, paying attention to what they emphasized and what they played down when they spoke about me, and this helped me fine-tune my discourse" (I25).

The excerpts from these interviews illustrate the socially constructed nature of storytelling identity work. These and other interviewees stated that the verbal and nonverbal reactions from other significant individuals prompted them to engage in a state of sympathetic introspection, leading them to critically reflect on how others would react to their personal storytelling. Thus, the data allowed us to identify that the more the interviewed CEOs considered feedback and learned from the personal storytelling of their peers, the more effectively they were able to construct narratives that they and others believed in. Therefore, we theorize that:

Proposition 3: Storytelling identity work is more likely to generate higher levels of intra and intersubjective validation when new CEOs co-construct their narratives through interactions with other CEOs and other significant members of the audience.

The second factor identified as contributing to the perception of intra and intersubjective validation in storytelling identity work was the participants’ realization that their storytelling led them to feel “sufficiently authentic” and “sufficiently impressive” during their identity transition processes. Several participants reported a paradox between “feeling loyal to what happened” (I6) and “impressing people” (I42). In this process, it was recurrently mentioned that the pursuit of extreme authenticity, where one truthfully recounts everything that occurred in a given event, tends to lead to the failure of personal storytelling. The same applies to the pursuit of exaggerated levels of impression management, where there are excessive attempts to captivate the audience. Between these extremes, there is a “more reasonable level of loyalty to what actually happened and the need to be approved” (I24). Thus, several participants, as exemplified below, demonstrated an understanding that to achieve intra and intersubjective validation, stories should not be overly authentic or excessively impressive. Instead, they should be what we categorize as “sufficiently authentic” and “sufficiently impressive,” both *in vivo* codes found in the data.

"I observed the following: I don't like lying, I don't like exaggerating, I'm very much focused on facts, and I find this artificial storytelling that I see out there quite ridiculous. But I realized that I needed to do up a little bit, otherwise, no one would believe me. So, I started revisiting the way I talked about my trajectory at [previous company] and added a subtle exaggeration here and there. But it was subtle enough for me to still feel authentic while managing to captivate the audience. It's a balancing act." (I23)

"You can't exaggerate in the story. It has to be surprising, it has to have that CEO presence, but if you exaggerate too much in your accomplishments, it becomes false and then it's worse, it becomes a joke. But if you're not willing to make those adjustments, if you want to be completely faithful to what happened in your life, everything becomes dull, and you don't reach that mythical figure that a CEO has to embody. So you have to be sufficiently authentic and sufficiently impressive. The key is finding a middle ground." (I22)

Thus, we suggest that:

Proposition 4: Storytelling identity work tends to generate greater feelings of intra and intersubjective validation when new CEOs are able to, through the stories they tell, experience (a) sufficient authenticity and (b) sufficient impressiveness during their identity transition processes.

### 5.3. What causes stories to be revised or retained in the self?

According to the accounts of the interviewees, some stories they told became part of their repertoire, while others were discarded over time. In the third research question that guided the construction of our grounded theory, we sought to explain what led to this variation. Because of what was proposed in P4, the success achieved with a story by feeling “sufficiently authentic” and “sufficiently impressive” led certain self-narratives to be repeated and incorporated into the self. Several interviewees mentioned that a story proved to be “sustainable,” “viable,” and “plausible” when it met these criteria, which resulted in its retelling and subsequently shaping and reflecting their self. The following account supports this finding.

Interviewer: “What happened over time with the stories that had this middle ground between feeling sufficiently authentic and sufficiently impressive?”

Interviewee: “They ended up being retold because I saw that they worked, they were viable. It's a story that passed the natural selection of life and became part of my movie. It became a scene in my movie. The movie that represents who I am, who I feel I am, and who people see me as” (I22).

As evidenced in this account, individuals possess a set of stories they tell about themselves, which shape and reflect how they define themselves and how such self-definition is socially validated. This repertoire of stories is categorized here as an “identity narrative.” Therefore, in order to be retained in the self, a story must be interpreted as reaching sufficient levels of fidelity to the perception of the events that occurred (being authentic) and to the need to meet expectations regarding the CEO role (being impressive). Considering this, we theorized that:

Proposition 5: When new CEOs engage in storytelling identity work, such stories are more likely to be retained in their identity narrative when they enable them to feel (a) “sufficiently authentic” and (b) “sufficiently impressive.”

Another factor has also proved relevant in the process of retaining stories within the identity narrative of new CEOs: coherence with the other stories intentionally or unintentionally told by the same individual throughout their life. According to the accounts of research participants, new CEOs have a history of stories shared during the early months of their recent role and throughout their professional journey. Many of these stories are known to their interactants, whether through “media interviews, YouTube videos, stories retold by others, or even posts on social media platforms such as Instagram and Facebook” (I41). Some of these stories were intentionally shared, such as those included in “recorded institutional speeches” (I42) and “other stories that used to be told in previous companies” (I45), while others represent unintentional revelations of identity, such as old social media posts. When the story being told is perceived as coherent with the preexisting identity narrative (whether intentional or not), it tends to be retained in the self. However, when the new CEO realizes that the audience interprets the story as conflicting with elements (e.g., archetype, moral of the story) of their identity narrative, it tends to be discarded. The following evidence illustrates this finding:

"I am like a book, you know? There are plenty of stories out there. I supported Bolsonaro [former President of Brazil] in the 2018 elections and made some right-wing posts against feminism that I didn't even remember. But when I took on this position, I realized that the company has a pro-women stance, and the previous CEO was a woman. So, I started portraying myself as a supporter of the cause, you know? But people took screenshots, it ended up being shared in many places, and there was a big uproar. I found out what it means to be canceled! Then I made a statement, saying that I've changed, but I had to soften this new version because it wasn't sticking. However, when I told stories about achieving targets, doubling results when I was a sales director, everyone believed me. There were plenty of witnesses of these achievements, so I could even embellish the stories a bit here and there, just lightly." (I1)

Therefore, we present the proposition of our model:

Proposition 6: When new CEOs adopt storytelling identity work, such stories tend to be retained in their identity narrative when they are perceived as consistent with other stories told (a) intentionally and (b) unintentionally by the same individual throughout their life.

## 6. Discussion

This study contributes to advancing the understanding of the relationship between role transitions and identities (Currie et al., 2010; Hennekam, 2016; Bordia et al., 2020) in five main ways. First, the concept of identity work has been widely discussed in the literature, and numerous studies have theorized how individuals construct and revise their selves in the workplace (Li et al., 2023; Sentuti et al., 2023; van Merriënboer et al., 2023). However, this study innovates by highlighting the role of storytelling in the process of constructing and expressing the self in the context of work. This contribution becomes even more significant in the case of new CEOs, given the high importance of this specific type of macro work role transition for both the lives of these professionals and the organizations they lead (Krishnan Kozhikode and Krishnan, 2022; Nguyen and Lee, 2023).

Second, previous studies have explored different forms of identity work. More specifically, it is possible to highlight modes of identity work grounded in cognitive aspects (Brown, 2017; Carollo and Guerci, 2017), physical aspects (Byron and Laurence, 2015; Felix and Cavazotte, 2019), behavioral aspects (Kreiner et al., 2006; Muhr, 2012; Conde et al., 2023), and discursive aspects (Clarke et al., 2009; Gomes and Felix, 2019) through which individuals seek to negotiate their selves. Although these modes are not mutually exclusive, the studies mentioned in this paragraph tend to focus on one of them. In the case of the present study, there is a clear emphasis on discursive aspects of identity work. However, while previous studies that fall within this perspective have highlighted elements such as lies, humor, metaphors, and bantering as ways to reinforce desired identities (Alvesson, 1998; Leavitt and Sluss, 2015; Carollo and Guerci, 2017; Huber and Brown, 2017), we emphasize the role of storytelling. Thus, we contribute to expanding the literature’s repertoire on how individuals use discourse to construct a desired self in the context of work.

Third, previous studies have already explored the trial-and-error process of constructing provisional selves through storytelling, which, although essential, is also seen as risky (Corlett et al., 2021). This is because any deviation in the CEO’s self-expression can be detrimental to both them and the company they lead (Byrne et al., 2021). However, this study delves deeper into how individuals may seek to manage the paradox between managing others’ impressions and feeling validated by their audience. In contrast to the binary view often found in the literature, the present study responds to the call for research that seeks to understand the nuances involved in the process of self-expression and audience enchantment that is often experienced by new CEOs (Ibarra, 2015; Verhaal and Dobrev, 2022). While previous studies have explored how these individuals seek to influence the impression of their audience in strategic presentations (Whittington et al., 2016; Collewaert et al., 2021), letters (Boudt and Thewissen, 2019), financial reports (Bujaki and McConomy, 2015), and online social networks (Zhang and Mooney Murphy, 2021), this study does so from the perspective of storytelling. The notion of feeling “sufficiently impressive” holds promise for encouraging a more nuanced view in the CEO’s identity work process.

Fourth, this study also encourages overcoming a binary view of authenticity in cases of macro role transitions. The literature on this topic predominantly interprets authenticity as the result of alignment between individuals’ external and internal identity (Caza et al., 2018; Bailey and Iyengar, 2022; Boytos et al., 2022). This interpretation suggests that individuals have an absolute awareness of who they are and how to express their identities, although recent studies challenge this notion (Wilt et al., 2019; Hennekam and Ladge, 2022). The present study aligns with this emerging stream of research (O’Neil et al., 2020; Hennekam and Ladge, 2022) that questions the view that individuals express a “true self” at work (Peterson, 2005). In the specific case of this study, a non-binary view of authenticity was explored from the perspective of new CEOs. We argue that this approach opens relevant research possibilities for understanding the shades of gray present in authenticity involved in CEOs’ identity management.

Fifth, this study bridges the literature on storytelling and identity work in the workplace and theorizes about processes that are important for the professional lives of CEOs but extend to their very constitution as individuals (Ibarra and Barbulescu, 2010; Bloom et al., 2021). Specifically, although not a longitudinal study, the research collected retrospective data over time, enabling a processual theorization of the phenomenon of identities in the workplace (Haynie and Shepherd, 2011). Given that the dynamism of the contemporary market demands increasingly careful adjustments in professionals’ self-presentation (Mansur and Felix, 2021), particularly those in strategic positions (Bach and Serrano-Velarde, 2015; Byrnes and Taylor, 2015), such a processual perspective becomes essential for a broader understanding of the experience of new CEOs.

## 7. Final remarks

### 7.1. Conclusion

In this study, we sought to explain: (a) under what conditions the phenomenon of CEO identity storytelling tends to occur; (b) what characterizes the success of storytelling identity work; (c) what leads to the revision or retention of stories in the self. The results indicated that storytelling identity work is employed by new CEOs during their transition period to this role, especially when their new position involves higher levels of visibility and a change in prestige compared to their previous role. They also suggested that storytelling identity work is more likely to be successful when the stories are co-constructed and validated with other significant individuals, and when they enable the new CEOs to feel “sufficiently authentic” and “sufficiently impressive.” Lastly, we theorized that these feelings, along with a sense of coherence between the story being told and the narratives expressed consciously or unconsciously by the interviewees throughout their lives, contributed to the retention of the story within the individual’s self.

### 7.2. Implications for practice

The theoretical model presented has implications for practice. For new CEOs, the study highlights the importance of utilizing storytelling identity work, especially in the early stages of assuming their new role. Specifically, the study emphasizes the risks of excessive candor, and of disregarding the need to manage audience impressions in the pursuit of authenticity, which can lead to rejection from other stakeholders. Additionally, it demonstrates that the opposite behavior of compromising one’s sense of authenticity in favor of an excessive desire to impress the audience can also result in social invalidation. Therefore, despite external pressures to create narratives that captivate and mobilize followers and other members of society, and the need to express oneself in their new role, it is necessary to avoid extreme behaviors that tend to become unsustainable in the long run.

Thus, the model presented suggests that authenticity and impression management are not binary concepts and should be understood as nuanced shades of gray. Based on this finding, we suggest that individuals engage in an iterative process of experimentation to find a viable level of alignment in their stories, where they feel a sufficient level of authenticity and effective impression management. In practical terms, this means challenging classical and polarized views that leaders must either seek their “true self” or adopt strategies that are not always well-considered for personal branding. Thus, we suggest that leaders view the dichotomy of “authenticity” and “social acceptance” not as a choice between mutually exclusive options, but as a paradox to manage.

Furthermore, the findings also highlight the importance of consistency among the different stories that new CEOs have told throughout their lives. When the CEO’s new company has behavioral expectations that differ from those exhibited by the professional over time, it becomes necessary to exercise caution so that the stories are not perceived as implausible and, therefore, not subject to social validation. The results also warrant recommending that these executives conduct a comprehensive review of the content posted on their social media platforms, including old posts, likes, tags, and comments from other individuals. In this review, unintended disclosure of identity should be avoided, as the nature of this communication channel often leads to forgetting information shared in the past that may be inconsistent with the current role expectations experienced in the present.

The study also provides suggestions for the practice of professionals working in organizations in support or oversight positions regarding the performance of CEOs. While the demands for authenticity are intrasubjective and, therefore, relate to individual issues of CEOs, the need to impress often reflects pressures from shareholders, marketing professionals, or mentors. Given the risks that our model highlights for the practice of storytelling identity work, we recommend that these professionals carefully assess the level of expectations they place on CEOs to represent the organization through their storytelling. This does not mean that it is not necessary or healthy to encourage CEOs to use stories to build positive narratives about themselves and the organization. However, the risks of this practice suggest caution and even the creation of a committee composed of career mentors, media trainers, and psychologists to help executives develop a strategy that allows them to tell tales about themselves that are simultaneously authentic and impressive.

### 7.3. Limitations and future research

This study has limitations that can be addressed through future research. First, we did not segment our analyses based on demographic characteristics of the interviewees. However, during the analysis process, we identified that the excesses in the pursuit of storytelling identity work varied according to the CEOs’ gender. While men more frequently reported engaging in excessive behaviors to impress others, women more frequently expressed discomfort in creating stories perceived as exaggerated about themselves. The data revealed a tendency suggesting that women may have a greater sensitivity to the discomfort of not feeling authentic. Thus, the degrees of sufficiency in authenticity and impression management appeared to vary according to the interviewees’ gender. Therefore, future studies are encouraged to explore gender differences in personal storytelling identity work in greater detail. To achieve this, it would be important to access samples that include a larger number of female CEOs.

Second, the study primarily involved Brazilian CEOs. Even in cases where the CEOs were not from Brazil, the interviews were conducted with individuals residing in this South American country. Brazil is known for its relational culture, high power distance, and the need to demonstrate power as a means of gaining social prestige (Hofstede et al., 2010; von Borell de Araujo et al., 2014). Therefore, it is possible that these cultural characteristics of Brazil may have influenced certain elements of the model, such as a greater need to impress the audience than what might be found in other countries. Consequently, studies could explore cross-cultural differences in the use of storytelling identity work among CEOs from different countries who also interact with individuals from different cultures. Additionally, it would be interesting to theorize about the differences in experiences among CEOs who interact in national, regional, or global contexts.

Third, the analysis remained limited to an individual level, but the data made it clear that certain storytelling practices are not solely the result of individual choices but rather of an institutional isomorphic script. Future studies could explore in greater detail the effects of inter-organizational transitions of CEOs who assume this role in sectors where there are conflicting institutionalized scripts regarding personal storytelling by leaders. Furthermore, given the dynamic nature of social expectations regarding the role of CEOs (Bach and Serrano-Velarde, 2015; Byrnes and Taylor, 2015), subsequent research could also investigate how scripts in terms of personal storytelling evolve over time within the same sector.

## Data availability statement

The raw data supporting the conclusions of this article will be made available by the authors, without undue reservation.

## Ethics statement

The studies involving humans were approved by Fucape Business School Ethics Comittee. The studies were conducted in accordance with the local legislation and institutional requirements. The participants provided their written informed consent to participate in this study.

## Author contributions

BF and RS has collected all the data and wrote the manuscript. BF, RS, and AT analyzed the data. All authors contributed to the article and approved the submitted version.
